# Uncovering Sub-Structure and Genomic Profiles in Across-Countries Subpopulations of Angus Cattle

**DOI:** 10.1038/s41598-020-65565-1

**Published:** 2020-05-29

**Authors:** Diercles Francisco Cardoso, Gerardo Alves Fernandes Júnior, Daiane Cristina Becker Scalez, Anderson Antonio Carvalho Alves, Ana Fabrícia Braga Magalhães, Tiago Bresolin, Ricardo Vieira Ventura, Changxi Li, Márcia Cristina de Sena Oliveira, Laercio Ribeiro Porto-Neto, Roberto Carvalheiro, Henrique Nunes de Oliveira, Humberto Tonhati, Lucia Galvão Albuquerque

**Affiliations:** 1 Department of Animal Science, School of Agricultural and Veterinarian Science, São Paulo State University (UNESP), Jaboticabal, SP Brazil; 2 Department of Animal Nutrition and Production, School of Veterinary Medicine and Animal Science (FMVZ), University of Sao Paulo (USP), Pirassununga, SP Brazil; 3 Department of Agricultural Food and Nutritional Science, Faculty of Agricultural, Life & Environmental Sciences, University of Alberta, Edmonton, AB Canada; 4 Lacombe Research and Development Centre, Agriculture and Agri-Food Canada, Lacombe, AB Canada; 5 Embrapa Pecuária Sudeste, São Carlos, SP Brazil; 6 CSIRO Agriculture & Food, 306 Carmody Rd., St. Lucia, Brisbane, QLD Australia; 7 National Council for Science and Technological Development, Brasília, Distrito Federal Brazil; 8 Present Address: Centre for Genetic Improvement of Livestock, Department of Animal Biosciences, University of Guelph, Guelph, ON Canada

**Keywords:** Comparative genomics, Population genetics, Genetic variation, Animal breeding

## Abstract

Highlighting genomic profiles for geographically distinct subpopulations of the same breed may provide insights into adaptation mechanisms to different environments, reveal genomic regions divergently selected, and offer initial guidance to joint genomic analysis. Here, we characterized similarities and differences between the genomic patterns of Angus subpopulations, born and raised in Canada (N = 382) and Brazil (N = 566). Furthermore, we systematically scanned for selection signatures based on the detection of autozygosity islands common between the two subpopulations, and signals of divergent selection, via F_ST_ and varLD tests. The principal component analysis revealed a sub-structure with a close connection between the two subpopulations. The averages of genomic relationships, inbreeding coefficients, and linkage disequilibrium at varying genomic distances were rather similar across them, suggesting non-accentuated differences in overall genomic diversity. Autozygosity islands revealed selection signatures common to both subpopulations at chromosomes 13 (63.77–65.25 Mb) and 14 (22.81–23.57 Mb), which are notably known regions affecting growth traits. Nevertheless, further autozygosity islands along with F_ST_ and varLD tests unravel particular sites with accentuated population subdivision at BTAs 7 and 18 overlapping with known QTL and candidate genes of reproductive performance, thermoregulation, and resistance to infectious diseases. Our findings indicate overall genomic similarity between Angus subpopulations, with noticeable signals of divergent selection in genomic regions associated with the adaptation in different environments.

## Introduction

Angus is a taurine breed (*Bos primigenius taurus*) of moderate frame size, originated in the Highlands of Scotland. It has become adopted worldwide for beef production, due primarily to its distinguished carcass and meat quality highly attractive in currently demanding market^1^. The introduction of Angus in Canada and Brazil occurred during the 19^th^ and early 20^th^ centuries, respectively. In both countries, black and red coat color variants have been registered in a single herd book^2,3^. In Canada, purebred Angus is found throughout all provinces, being the most popular beef breed, in terms of number of new registered animals per year (56,003 in 2019)^4,5^. In Brazil, nearly 51% of 9.62 million beef cattle semen doses commercialized in 2018 were from Angus breed, having more than half of the semen straws imported from Canada and the U.S.^6^. Despite widely importing foreign genetic material, Brazil also performs national genetic evaluation including Angus purebred local records, which allows the identification of superior Brazilian sires. The progeny originated from locally selected animals tend to be more adaptable to tropical conditions and more robust to environmental changes^7^.

Combining international data of the same breed with the employment of proper adjustment factors has long been referred to as a promising strategy improving accuracies for cattle genetic analysis^8–10^, including the Angus breed^11^. Genomic evaluation also benefits from this data combining strategy as dense SNP genotypes will overcome the lack of common ancestry between subpopulations when the pedigree is not deep enough^12^. The genomic prediction relies on the linkage disequilibrium (LD) between the genotyped markers and quantitative trait *loci* (QTL), which may not persist between subpopulations, or may even be in reversed phase. Although it is expected that LD phases extend across large distances for subpopulations of the same breed in different countries^13^, this information is yet to be adequately assessed in Angus cattle.

The geographic division of Angus subpopulations may increase the diversity and sub-structure within the breed, albeit they originated from a unique domestication center. Since, differentiation in allelic frequencies, haplotypes diversity and LD tend to increase over time due to reduced common ancestry between subpopulations, different environmental pressures, and potential differences in selection schemes^13,14^. Furthermore, selection, either natural or human-oriented, intensifies the differentiation between subpopulations by driving particular genomic regions towards special patterns in each specific geographical location^15^.

Here, we characterize differences and similarities in the genomic profile of Angus from Canada and Brazil, by assessing the genomic level of relationship, inbreeding, linkage disequilibrium and persistence of linkage phase in both subpopulations. In addition, we scan for selection signatures that may shed light on the molecular basis of selected traits and divergent adaptation mechanisms on the two subpopulations. Although the current study has been focused on the population genomic aspects, our results are relevant to genomic predictions, especially to further collaborative analyses incorporating the studied herds.

## Results

### Population substructure and relationship

Here, we evaluated a sample of 948 Angus cattle (382 from Canada and 566 from Brazil) with genotypic information of 31,483 autosomal markers, spanning up to 2.5 GB of the bovine genome in an average spacing of 78.6Kb (ranging from 0.006Kb to 3,049Kb). The overall averages of observed heterozygosity (0.353 ± 0.012; 0.350 ± 0.009) and minor allele frequency (MAF: 0.268 ± 0.141; 0.263 ± 0.144) did not differ between Brazilian and Canadian subpopulations (Table 1). The genomic relationship between samples ranged from 0.5 to 0.81 and the average estimated for pairs of individuals from different subpopulations was 0.577 ± 0.021. The average genomic relationship within the Canadian subpopulation was slightly higher than the Brazilian average (Table 1).

**Table 1:** Averages and standard deviations of minor allele frequency (MAF), observed heterozygosity (HET), genomic relationship and inbreeding derived from genomic relationship matrix (F_GRM_) and runs of homozygosity (F_ROH_) in Angus subpopulations.

Subpopulations	MAF	HET	Genomic relationship^a^	F_GRM_	F_ROH_
General	0.268 ± 0.014	0.352 ± 0.010	0.587 ± 0.035	0.296 ± 0.022	0.144 ± 0.027
Brazilian	0.268 ± 0.141	0.353 ± 0.012	0.590 ± 0.041	0.294 ± 0.024	0.139 ± 0.030
Canadian	0.263 ± 0.144	0.350 ± 0.009	0.613 ± 0.040	0.300 ± 0.017	0.152 ± 0.022

^a^Average of relationship between samples from different subpopulations (Brazilian vs. Canadian): 0.577 ± 0.021.

**Figure 1:**
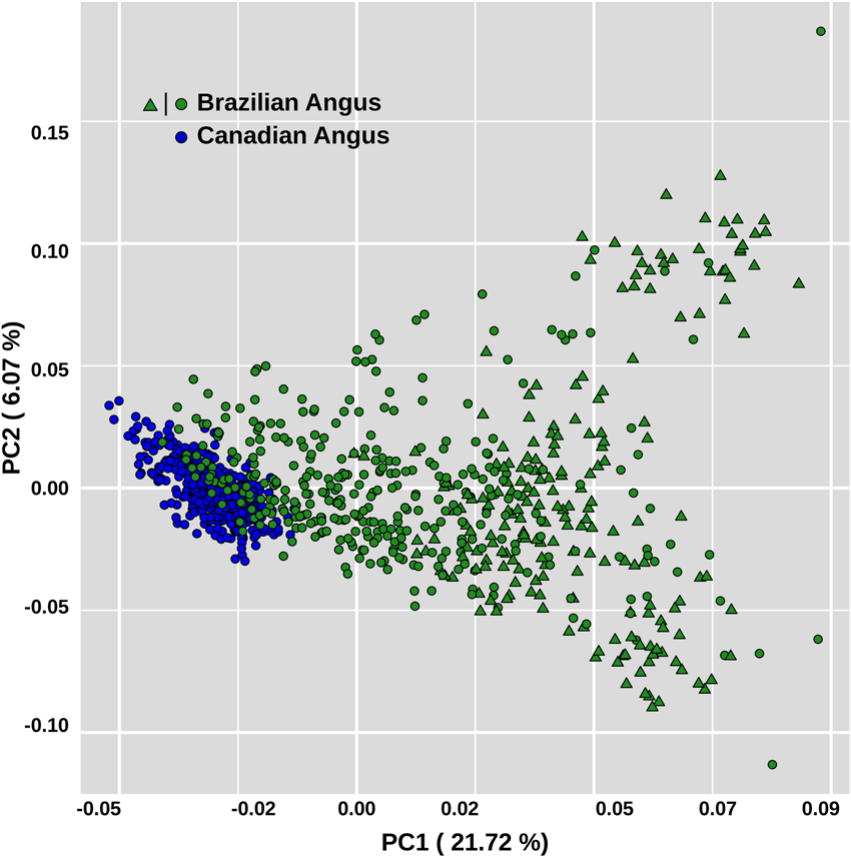
Principal component analysis of relationship matrix for Brazilian and Canadian Angus subpopulations. Blue and green circles represent black coat-color samples from Canada and Brazil, green triangles represent red samples from Brazil.

The proportion of variance in genomic relationship matrix (GRM) explained by the first and second principal components was 21.72% and 6.07%, respectively (Fig. 1). In line with the genomic relationship averages, principal component analysis (PCA) revealed greater diversity within the Brazilian samples than within Canadians and depicted two closely connected clusters representing the subpopulations. Although the Brazilian subpopulation included black and red coat colored samples, a clear extra population substructure was not observed within this subpopulation due to coat color variation (Fig. 1). It is noteworthy that the substructure that allowed distinguishing the two clusters representing the Angus Brazilian and Canadian subpopulations is not comparable with the stratification level involving different breeds (Supplementary Fig. S1). The level of differentiation between the two subpopulations, measured through the average of Wright’s F_ST_ statistics, was equal to 0.072 ± 0.021.

**Figure 2:**
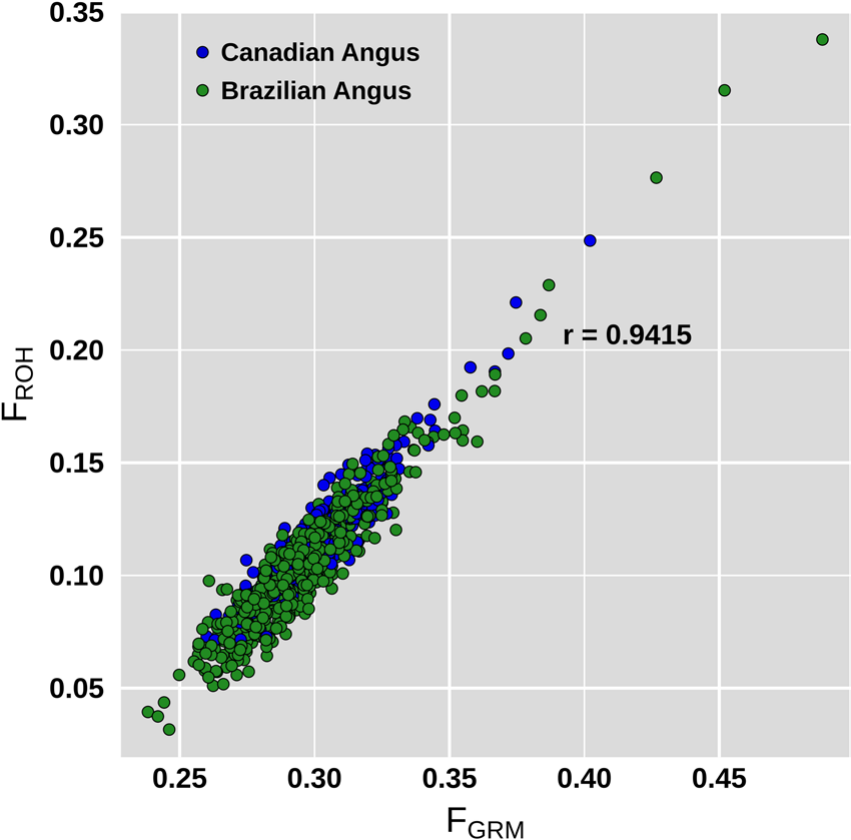
Genomic inbreeding estimated through the diagonal of the genomic relationship matrix (F_GRM_) and runs of homozygosity (F_ROH_) in the Angus subpopulations. Green and blue dots represent Brazilian and Canadian samples.

Averages of genomic inbreeding estimated using diagonal elements of GRM and runs of homozygosity (ROH) were similar in the two subpopulations (Table 1), being slightly higher in the Canadian. The correlation between these two metrics was equal to 0.9415, with F_GRM_ estimates being overall higher than F_ROH_ (Table 1, Fig. 2). Table 2 summarizes the distribution of ROH lengths by subpopulations. The mean length of ROHs and the proportion of segments longer than 8 Mb were higher in the Brazilian subpopulation. All samples presented at least one ROH segment longer than 8 Mb. In total, 59 and 63% of samples in the Canadian and Brazilian subpopulations, respectively, presented at least one ROH segment longer than 16 Mb.

**Table 2:** Summary of the length of runs of homozygosity (ROH) in two subpopulations of Angus Cattle.

Subpopulations	N^a^	Mean length ± SD (Mb)^b^	Min. (Mb)^c^	Max. (Mb)^c^	>4 Mb (%)^d^	>8 Mb (%)^d^	>16 Mb (%)^d^
Brazilian	28,020	5.088 ± 4.389	1.004	82.575	46.76	13.22	2.58
Canadian	21,910	4.873 ± 3.754	1.022	76.254	46.17	12.06	1.72

^a^Total number of ROH segments per subpopulation; ^b^Mean length of ROH and standard deviation (SD) per subpopulation; ^c^Minimum and Maximum observed length of ROH; ^d^Percentage of ROH segments longer than 4, 8 and 16Mb.

### Linkage disequilibrium

We used phased haplotypes to estimate linkage disequilibrium (LD), through r^2^ statistic. The number of SNPs, averages of SNP distances and r^2^ of adjacent SNPs per chromosome are presented in Supplementary Table S1. The r^2^ of all pairwise adjacent markers averaged 0.20 and 0.21 in the Brazilian and Canadian subpopulations, respectively (Table 3). The r^2^ average decreased according to the increase in distance classes of adjacent markers and increased according to the increase in the MAF threshold (Table 3). There was a smaller increase in r^2^ by rising the MAF threshold from 0 to 1%, than rising it from 1 to 5%.

**Table 3:** Average and standard error r^2^ of adjacent SNPs at various distance classes in the autosomal genome of Angus subpopulations.

Markers^a^	Subpopulations							
	Brazilian				Canadian			
	Adj^b^.	100Kb^c^	300Kb^c^	500Kb^c^	Adj^b^.	100Kb^c^	300Kb^c^	500Kb^c^
MAF > 0%	0.196 ± 0.001	0.218 ± 0.001	0.126 ± 0.002	0.101 ± 0.007	0.219 ± 0.001	0.243 ± 0.002	0.148 ± 0.002	0.120 ± 0.007
MAF > 1%	0.203 ± 0.001	0.227 ± 0.001	0.131 ± 0.002	0.105 ± 0.006	0.243 ± 0.001	0.244 ± 0.001	0.149 ± 0.003	0.121 ± 0.008
MAF > 5%	0.220 ± 0.002	0.251 ± 0.002	0.145 ± 0.002	0.122 ± 0.006	0.239 ± 0.002	0.269 ± 0.002	0.163 ± 0.002	0.141 ± 0.007

^a^With no criterion of MAF (MAF > 0%), MAF of 0.01 and MAF of 0.05 the exact number of SNPs were 31,483, 30,657 and 28,048, respectively.

^b^Average r² pairwise analyses considering 31,454, 30,628 and 28,019 combinations of adjacent SNPs, independent of the distance between SNPs.

^c^Average r² for adjacent SNPs spaced up to 100 Kb, 300 Kb and 500 Kb. The exact number of pairwise of SNPs in this classes were 24,124, 6,708 and 502 with MAF > 0%; 23,144, 6,816 and 539 with MAF > 1%; 20,211, 6,969 and 672 with MAF > 5%.

**Figure 3:**
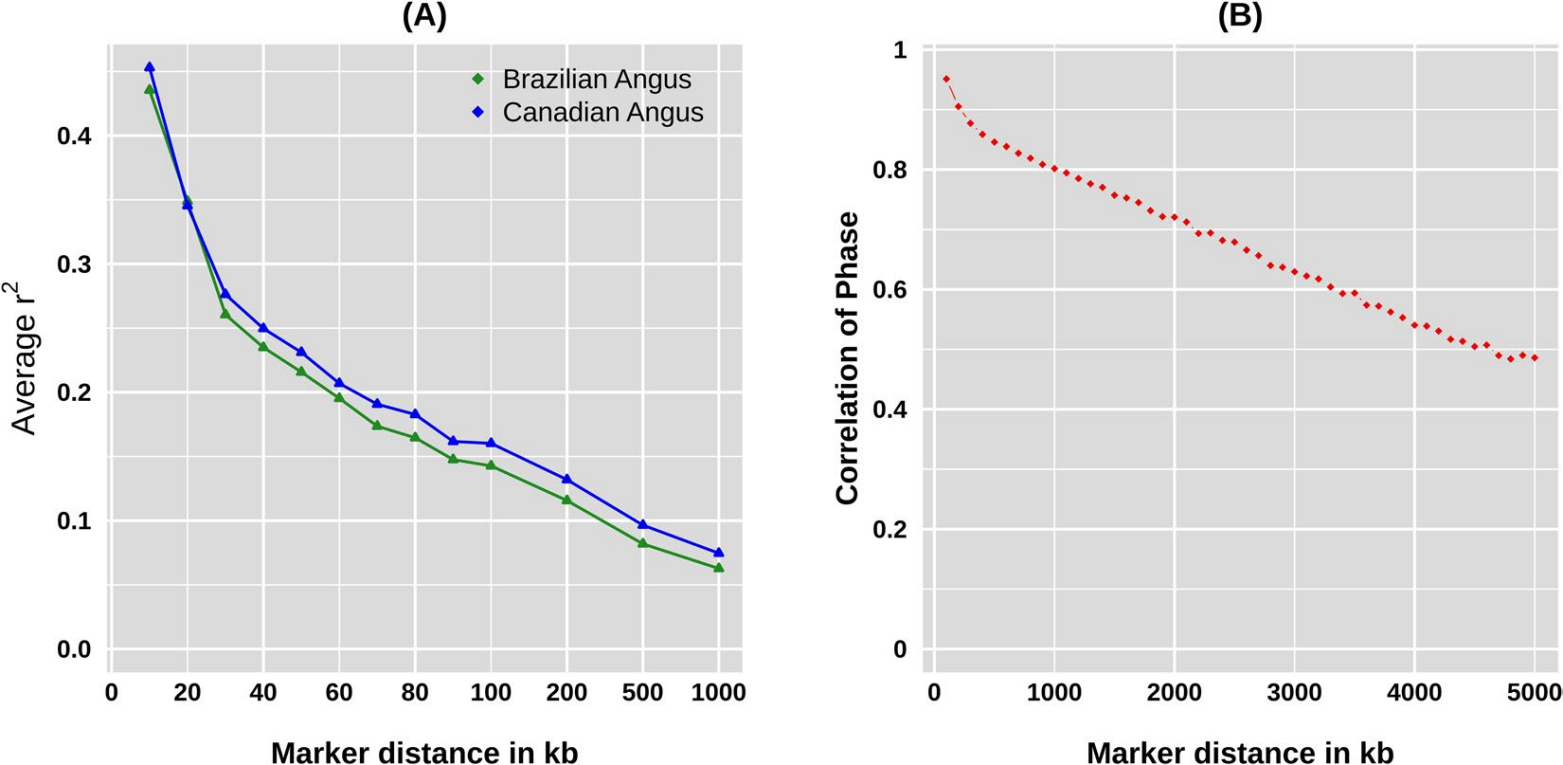
LD decay as a function of inter-marker distance (**A**) and correlation of linkage phase between Angus subpopulations for SNP pairs at varying distances (**B**).

With all combinations of syntenic SNPs, the overall average of r² kept higher than 0.2 for markers distanced up to 50Kb in both subpopulations (Fig. 3A). At distances around 1 Mb, the markers pairwise had r² averages equal to 0.06 and 0.07 in the Brazilian and Canadian subpopulations, respectively. Although the LD averages in the Canadian subpopulation were the largest for almost all distance classes, the slope of decay were consistent between the two subpopulations. The persistence of the phase between the two subpopulations remained high at a scale of nearly 0.50 up to a distance of 5 Mb (Fig. 3B) indicating agreement between linkage phases in the two subpopulations.

### Candidate regions under selection within and across subpopulation

**Figure 4:**
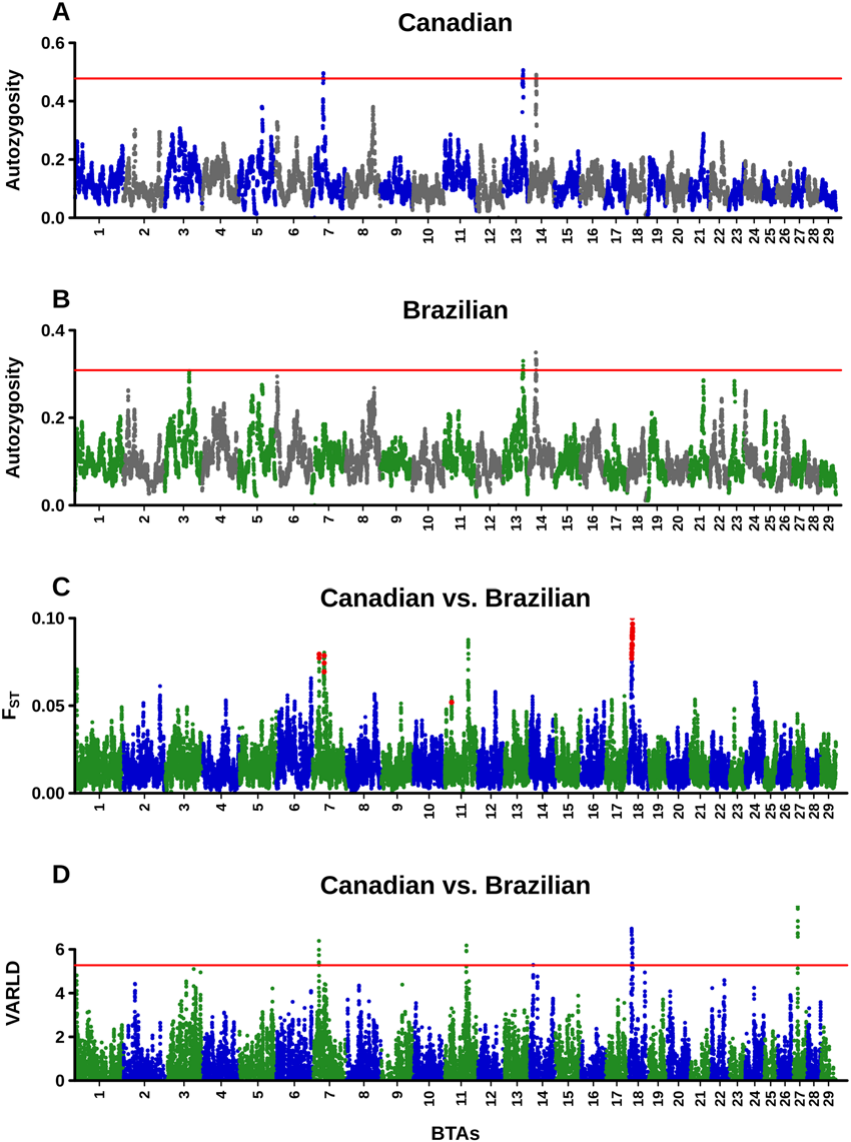
Genome-wide autozygosity, F_ST,_ and varLD scores. (**A**,**B**) Autozygosity scores per SNP within Brazilian and Canadian subpopulations. (**C**,**D**) F_ST_ and varLD scores averaged for 15 SNPs windows. Red lines indicate the cutoff of 99.9 percentile to ROH and varLD scores. Red dots represent F_ST_ windows in 99.9 percentile of their heterozygosity bins.

The ROH scan revealed 3 and 2 regions with at least two consecutive SNPs with autozygosity score above the empirical threshold (99.9 percentile) in the Canadian and Brazilian subpopulations, respectively (Fig. 4, Supplementary Tables S2). The F_ST_ and varLD tests (between-population comparative methods) identified 5 regions each, with at least two consecutive windows exceeding the empirical threshold (Fig. 4, Supplementary Tables S3). The length of these regions ranged from 160 Kb to 1910Kb and presented some overlapping between them. Table 4 lists 6 selection signatures, which represent genomic regions concordant between the 99.9 percentile of at least two of the independent tests, hence more likely to be true positive signals^16^.

**Table 4:** Genomic regions and candidates genes depicted as selection by combined results of ROH islands, F_ST_ and varLD tests in Brazilian and Canadian Subpopulations of Angus.

Genomic Regions^a^	Method	Population^b^	Genes^c^	Gene Ontology^d^
BTA7:21.31–21.89	varLD and FST	“BRA *vs*. CAN”	*IRF1* *KIF3A* *IL4*, *IL5*, *IL13*	Regulation of immune response Epidermis development Immune response
BTA7:37.84–38.64	ROH and F_ST_	“CAN” and “BRA *vs*. CAN”	*ARL10*, *UNC5A*	Integral component of membrane
BTA13:63.77–65.25	ROH	“BRA” and “CAN”	*MYH7B* *GDF5*	Myosin complex Growth factor activity
BTA14:22.81–23.57	ROH	“BRA” and “CAN”	*XKR4* *PLAG1*	Anatomical structure development Multicellular organism growth
BTA18:11.75–11.94	varLD and F_ST_	“BRA *vs*. CAN”	*IRF8* *COX4I1*	Immune response Integral component of membrane
BTA18:13.76–15.40	varLD and F_ST_	“BRA *vs*. CAN”	*CYBA* *MC1R* *DNAJA2*	Innate immune response Pigmentation Response to heat

^a^Chromosome:StartPosition(Mb):EndPosition(Mb);

^b^“BRA” and “CAN” represent ROH islands detected in the Canadian and Brazilian subpopulation, respectively; “BRA vs. CAN” signals of divergent selection;

^c^Only the most plausible candidate genes are shown;

^d^One of the ontologies of gene(s).

Two ROH islands common to both subpopulations were detected at BTA13:63.77–65.25 and BTA14:22.81–23.57 (Fig. 4A,B), surrounding candidate genes of stature, muscle development and lipid metabolisms, such as *MYH7B* (myosin heavy chain 7B), *GDF5* (growth differentiation factor 5), *PLAG1* (PLAG1 zinc finger) and *XKR4* (XK related 4)^17–20^. A further ROH island exclusively detected in the Canadian subpopulation, BTA7:37.84–38.64, overlapped with a signal of divergent selection highlighted via F_ST_ (Table 4, Fig. 4A,C), indicating it as a region that has been targeted by selection only in the Canadian subpopulation. This genomic region encompassed several genes encoding integral components of membrane related to reproductive traits. The *ARL10* (ADP-ribosylation factor-like 10) gene is suggested to play important role in bovine pre-implantation embryo development^21^ and the *UNC5A* (unc-5 netrin receptor A) gene previously showed association with reproductive traits in taurine breeds, such as the number of insemination per conception, fertility index and the interval between first and last insemination^22^.

Selection signatures revealed by the overlaps of the between-population differentiation methods (F_ST,_ and varLD), at BTA7:21.31–21.89 and BTA18:11.75–11.94, are putatively related to adaptation into adverse conditions across the different environments (Table 4, Fig. 4C,D). The selection signature BTA7:21.31–21.89 contained several adaptive immunity-related genes, including the *IRF1* (interferon regulatory factor 1) and *IL4* (Interleukin-4) genes, which control the differentiation of naive T helper (Th) cells and activation of cell-mediated or antibody-mediated immune responses^23,24^. In addition, the *KIF3A* (Kinesin family type 2 member 3 A) gene, mapped at this region, is a candidate to innate tick-resistance typical of some breeds^25^. Beyond immunity-related genes, the selection signature BTA18:11.75–11.94 comprised the *COX4I1* (cytochrome c oxidase subunit 4I1) that is related to thermoregulatory efficiency. This gene represents a candidate gene to body temperature regulation, which was previously associated with cold tolerance^26^, and was up-regulated in the liver of cows experimentally submitted to cold-stress^27^.

A further selection signature identified via F_ST_ and varLD, at BTA18:13.76–15.40, was the longest signal detected in the current study and comprised the *MC1R* gene, major gene to coat color determination in Angus^28,29^. Interestingly, this signal was detected even when the selection signature scan was re-performed keeping only samples of black coat color in both subpopulations (Supplementary Fig. S2). Other genes of this region that are plausible of being causing this signal include *DNAJA2* (DnaJ heat shock protein family member A2) that is involved in cellular responses to heat stress^30^, and some key players of immune responses, such as *CYBA* (cytochrome b-245 alpha chain) and *CDK10* (cyclin dependent kinase 10)^31–33^.

The selection signatures common between Brazilian and Canadian subpopulation (BTA13:63.83–64.87 and BTA14:24.43–26.20) comprised 41 genes, whereas the regions representing signals of divergent selection (ROH islands exclusively detected in one of the two subpopulation, F_ST,_ and varLD signals) comprised 74 genes. Significant enrichment of particular terms or biological processes was not detected in any of these gene lists.

## Discussion

Accentuated differences between the overall genomic diversity of two populations allow two assumptions, being (1) recent gene flow or adaptive genetic variation in the subpopulation of higher diversity, or (2) genetic drift, reduced effective population size or increased inbreeding in the population of lower diversity^34,35^. Although the genomic diversity patterns of the two subpopulations studied here were quite similar, the Brazilian samples showed higher heterozygosity, broader dispersion in PCA and lower averages of genomic relationship, inbreeding, and linkage disequilibrium than Canadian samples. The slightly lower diversity observed in the Canadian subpopulation is most likely due to the sample origin and its commercial purpose. The Canadian genotypes were obtained from a single experimental herd of reduced effective population size, in comparison with Brazilian samples that represented commercial herds, more opened to the eventual adoption of imported semen.

The observed heterozygosity within each Angus subpopulation was in agreement with averages varying from 0.357 to 0.399, reported in taurine breeds genotyped with high-density SNP panels^34,36^, but higher than the average of 0.28 previously reported to 42 Angus samples^37^. Presumably, this difference is due to the greater number of genotyped individuals studied here. Similar to previous results_,_ the correlation between F_GRM_ (with base allele frequency fixed to 0.5) and F_ROH_ was high, with greater estimates obtained to F_GRM_ due to the non-distinction between IBS and IBD alleles as done by F_ROH_^38,39^. Long ROHs (>8,000 Kb) were observed in both subpopulations, indicating relatively recent relatedness within both of them. Autozygous segments longer than 8,000 Kb and 16,000 Kb indicate nearly 6 and 3 generations, respectively, since the common ancestor that gives origin to both haplotype copies^40^.

The population structure depicted in PCA showed a great overlap between the clusters of the Brazilian and Canadian samples. One reason for this continued genomic connection resides in the recent common ancestry, also evidenced by the relative high LD phase persistence across the subpopulations. This recent common ancestry is most likely due to the importation of semen from Canada into Brazil, as well as the extensive importation of U.S. Angus sires into both countries (ASBIA 2018, http://www.asbia.org.br/wp-content/uploads/2018/10/INDEX-ASBIA-2017_completo.pdf).

The averages of r² considering all SNP pairs spaced up to 50Kb showed agreement with estimates previously reported for taurine breeds (r² ~ 0.25)^41,42^ and were higher than averages previously reported for indicine and taurine-indicine cross-bred cattle (r² ~ 0.18)^41,43^. The LD is a decisive parameter to define the required markers density for successful genomic prediction and genome-wide association studies^10,44^, being suggested that r² values of 0.2 are required to achieve high prediction accuracies (>0.8)^45^. Considering the bovine genome size of 2,875 Mb, and the averages of r² higher than 0.2 for adjacent SNPs spaced up to 100Kb in both Angus subpopulations, the minimum genotyping density required to these subpopulations would be 28,750 SNPs. The distribution of 31,483 SNPs common between two different commercial arrays maintained some gaps longer than 100Kb along chromosomes. Thus, the imputation of genotypes to the non-common SNPs would be a feasible strategy to enhance the marker density in both subpopulations, fulfilling long gaps and assuring great prediction accuracies in further studies. Furthermore, a single reference population genotyped with a higher density of markers could supply reference haplotypes to both subpopulations, due to the strong preserved phase between these two subpopulations.

The persistence of phase across populations may indicate allele-QTL preserved phases, being another very important issue to joint-genomic analysis. Porto-Neto *et al*.^46^ reported considerable gains in genomic prediction accuracies in five cattle traits using only SNPs of consistent linkage phase between two distinct populations. The linkage phase correlation between the Brazilian and Canadian subpopulations stayed high for genomic distances extended to 5 Mb, corresponding well with estimates of approximately 0.7 reported for subpopulations of a single breed^42^. This persistence was significantly higher than estimates involving different breeds (approximately 0.45)^41^. Therefore, combining the two subpopulations studied here to perform genomic prediction would result in gains in reliability, as it would enable increased reference population without the effect of SNPs in reversed phase masking the marker-QTL association.

The suitability of ROH islands for the selection signature scanning was reinforced here, due to several overlaps with QTL of traits undergoing selection and previously reported selection signatures of beef cattle. The two ROH islands found common between the Brazilian and Canadian samples suggested common selection events on BTA13 and BTA14, at genomic regions harboring genes known to be associated with stature in mammals^19,47,48^, thus putatively influential on growth traits undergoing selection in both subpopulations. These two signals were previously reported as selection signatures of Angus^49^ and were significantly associated with the body weight of Brahman^50^, in a study based on the reference UMD3.1. Reasonable candidate genes of BTA13 includes the *GDF5* gene, which belongs to a family of bone morphogenetic proteins that stimulate bone formation and regulate growth^51^, and *MHY7B* that is expressed in the skeletal muscle and downregulated in double-muscled breeds^20^. The region of BTA14 comprising *PLAG1* and *XKR4* genes is well-known for its pleiotropic effect on economically important traits of beef cattle, such as backfat thickness, ribeye area, body and carcass weight^52,54,55^, as well as the serum level of the growth-related hormones, IGF1^53^.

An impressive signal of genomic differentiation between the subpopulations was identified at BTA7, corresponding to the ROH island exclusive of the Canadian subpopulation that partially coincided with F_ST_ results. The rs110428791 SNP, which is an intronic variant of gene *UNC5A* mapped at this selection signature, was previously associated with reproductive performance in taurine breeds, in a study based on the reference genome UMD3.1^22^. In addition, homologous genes of *RNF44* (ring finger protein 44), and *UIMC1* (ubiquitin interaction motif containing 1), have been associated with reproductive risks and duration of reproductive life in women^56,57^. These findings suggest selection pressures in Canadian subpopulation with a balance between growth and reproductive traits.

The signal of divergent selection detected at BTA7:21.31–21.89 is most likely related to differences in the ability of quicker and strong response towards pathogen invasions across the subpopulations raised in different environments. This genomic region was previously identified as a QTL associated with resistance to viral load in Holstein cattle^58^, and it comprises genes that showed differential expression between susceptible and resistant hosts to cattle tick (*KIF3A*)^25^, mange infested and uninfested animals (*IL4*, *IL5* and *IL13*)^59^, healthy and with mastitis cows (*IL4*)^60^. In addition, this selection signature was associated with the adaptation of taurine breeds to harsh environments^61^ and of indicine breeds to not so harsh, but still challenging environmental conditions^62^.

Two highlighted regions of BTA18 overlapped with selection signatures previously reported in Russian taurine breeds adapted to harsh weather^61^ and harbored candidate genes to immune and thermoregulatory efficiency. It is noteworthy that the longest selection signature (BTA18:13.76–15.40), encompassed the major gene for coat color variation in Angus, *MC1R*^28^. We were able to refuse the likelihood of this signal being related to the presence of red Angus samples (recessive homozygous) in the genotyped Brazilian subpopulation. However, we didn’t estimate frequencies of recessive allele among the samples of black coat color, since our genotyping information did not include the causal mutation for pigmentation in Angus, rs109688013^29^. Nevertheless, none of the two countries perform selection based on coat colors. Thus, it is not discarded the hypothesis of this signal being caused by other genes in this region, related to better performances on selected traits under challenging environmental conditions. Furthermore, this region has been indicated as QTL of resistance to infection in dairy cattle^32,63^, and revealed as a selection signature of cattle breeds with coat color phenotypes not determined by *MC1R*, such as Brown Swiss, Hanwoo and Nguni^33,64,65^. Further investigation adopting denser panels or even sequencing data could narrow down this region and empower the identification of the causal mutation(s) behind this signal.

Despite the potential of parallel selection in promoting differentiation of geographically isolated subpopulations of the same breed^13^, the Angus subpopulation from Canada and Brazil still present substantial genomic connection likely due to common ancestry (mainly influenced by the use of semen from common sires) and similarity between the selection schemes, primarily focused on growth-related traits in both countries^7,66^. Nevertheless, signatures of divergent selection pinpointed particular loci, related to reproductive performance, thermoregulation, and resistance to infectious diseases. Therefore, specific population subdivision might have been caused by both adaptation to distinct environments and particularities of selection criteria applied in each country, such as an additional emphasis on breeding indexes that combines fertility and growth traits in Canada^67–69^.

## Methods

### Genotyping information

Care and Use Committee approval was not required for the current study since it was performed with previously genotyped samples.

The genotypic data consisted of 382 and 566 Angus born and raised in Canada and Brazil, respectively. The Canadian subpopulation, previously described in Chen *et al*.^70^, consisted of steers born at the Onefour Research Substation of the Agriculture and Agri-Food Canada Research Centre (AAFC) at Lethbridge - AB, from 2004 to 2008. These samples were genotyped for approximately 54 K SNPs included in the BovineSNP50 (Illumina, San Diego, CA) panel. The Brazilian subpopulation comprised 352 heifers born in 2013 at Santa Helena farm at Uruguaiana - RS, and 214 influential Angus sires of Brazilian herds. Brazilian samples were genotyped for approximately 139 K SNPs included on GGP-150K - NEOGEN (GeneSeek, Lincoln, NE) panel. The genotyped Canadian subpopulation was exclusively composed of black coat color samples, whereas 206 out of 566 Brazilian samples were red Angus.

All the genotyped samples presented call-rate higher than 0.90. The SNPs from both arrays were remapped according to the new bovine assembly, ARS-UCD 1.2^71^, using publicly available coordinates provided at https://www.animalgenome.org/repository/cattle/UMC_bovine_coordinates. SNPs presenting different positions among the different coordinate files aligned into the ARS-UCD 1.2 (N = 12) were filtered out from both arrays during the remapping process. Then, non-autosomal markers, markers presenting call-rate lower than 0.90 or showing a significant deviation from Hardy-Weinberg equilibrium (p < 10^−5^) were removed in both subpopulations datasets. Imputation of missing genotypes and haplotype phasing was performed per subpopulation using the Fimpute v3^72^. Finally, only non-monomorphic markers, common between the two panels were kept to further analyses (n = 31,483).

### Genomic relationships and inbreeding coefficients

Relatedness between all pairs of individuals was estimated using the GRM computed according to method 1 in VanRaden^73^, with base allele frequency fixed to 0.5^39,74^. Genomic inbreeding coefficients based on GRM (F_GRM_) were estimated by subtracting one from its diagonal elements. Runs of homozygosity based inbreeding coefficients (F_ROH_) were estimated as the sum of lengths of ROHs of each individual divided by the length of the genome covered by the SNPs^75^. The ROH discovery was conducted using PLINK v1.9^76^, by sliding a 30 SNPs window in one SNP interval throughout each autosomal chromosome. One heterozygous genotype was permitted per window to account for occasional genotyping errors. The minimum length of an ROH segment was set to 1,000Kb, where a density of at least 1 SNP every 120Kb and no marker-intervals longer than 1,000Kb were required, following the recommended criteria of smaller density genotypes^77^.

SNP autozygosity scores were calculated per subpopulation and expressed as a proportion of animals in which each SNP appeared in an ROH. Genomic regions harboring at least two adjacent SNPs falling into the 99.9^th^ percentile were assigned as ROH islands. The positions of the first and the last SNP of these regions were assumed as the start and end point of ROH islands.

To assess the influence of ROH detection using a fixed sliding window (approach implemented in PLINK software^76^) in our results, the ROH detection was repeated with an approach based on run detection in consecutive SNPs, implemented in the package detectRUNS^78^ of R software v3.4.4^79^. This procedure revealed F_ROH_ values highly correlated (0.98) with those estimated in PLINK^76^, and essentially the same ROH islands (Supplementary Fig. S3, Tables S4).

### Population substructure

Principal component analysis was applied to the GRM to investigate the population sub-structure. Additionally, we performed PCA after adding publicly available genotypes of Holstein^80^ and Nelore^81^ to compare the stratification level between the subpopulations of a single breed with the stratification across different breeds (Supplementary Fig. S1).

### Analyses of linkage disequilibrium

Phased haplotypes were used to estimate the LD extent and persistence of phase, applying publicly available scripts at https:// www.msu.edu/~steibelj/JP_files/LD_estimate.htm. A measurement of LD was estimated for all syntenic SNP pairs by the genotype squared correlation (r^2^), as proposed by Hill and Robertson^82^. The decay of LD was then analyzed in each subpopulation using r² averages within bins according to the physical distances of SNP pairs, including 10 Kb intervals starting from 10 to 100 Kb, and the intervals 100–200 Kb, 200–500 Kb and 500–1000 Kb. To assess the effect of MAF on LD, the r^2^ was calculated with SNPs of minimum MAF higher than 0.01 (N = 30,657) and 0.05 (N = 28,048) in both subpopulations.

The consistency of the linkage phase for pairs of *loci* across subpopulations was analyzed through genotype correlation (r_ij_). We estimated r_ij_ between all pairs of SNPs spaced for shorter intervals than 10 Mb in both subpopulations. Then, the persistence of phase was estimated within intervals of 100Kb, starting from 0.1 to 5 Mb, as Pearson’s correlation of r_ij_ for each subpopulation, as follows:

1 $${R}_{m,n}=\frac{{\sum }_{(i,j)\in p}({r}_{ij(m)}-{\bar{r}}_{(m)})({r}_{ij(n)}-{\bar{r}}_{(n)})}{{s}_{(m)}{s}_{(n)}}$$

where R_*m,n*_ is the correlation of phase between r_ij_ in the subpopulation m (Brazilian) and n (Canadian); ${\bar{r}}_{(m)}$ and ${\bar{r}}_{(n)}$ are the averages of r_ij_ within interval p in the subpopulation m and n, respectively; and s_(m)_ and s_(n)_ are the standard deviation of r_ij_ in the subpopulation m and n, respectively^83^.

Contrasts between regional patterns of LD of the two subpopulations were performed through the statistic varLD^84^, implemented in varLD software^85^. The varLD method consists of building symmetric LD matrices in each subpopulation, with r^2^ of all pairs of SNPs within windows defined based on a fixed number of markers, sliding in one SNP interval. Next, eigenvalues of each symmetric matrix are computed per subpopulation, and raw varLD scores assigned as the sum of absolute differences between ranked eigenvalues of the homologous matrices. Finally, each raw varLD score is compared against the distribution with all windows values. Since one of the premises of varLD statistic is the establishment of windows encapsulating the same number of SNPs across the genome^84^, we choose to define windows of 15 SNPs (mean length of 1095.9 ± 389.9 Kb), instead of windows with fixed length size. Genomic regions with at least two consecutive windows falling in the 99.9^th^ percentile of varLD were considered indicative of divergent selection. Preliminary analyses carried out using a range of SNP numbers (15, 20, 25 and 30) yielded consistent results related to the majority of signals, with the shortest boundaries of the detected signals associated with the windows of 15 SNPs (Supplementary Fig. S4).

### Allelic frequency differentiation

The proportion of total variance in allele frequencies due to differences between subpopulations was quantified by applying the Wright’s fixation index (F_ST_), estimated according to Cockerham and Weir^86^ using the “fsthet” package^87^ in R software, v3.4.4^79^. F_ST_ and heterozygosity were averaged in sliding windows set equal to those of varLD procedure (15 SNPs, sliding in one SNP interval). To control false positive discoveries in F_ST_ test the empirical thresholds were defined based on the F_ST_-heterozygosity outliers approach^87^. Windows were grouped into 20 bins according to heterozygosity and genomic regions were considered as putative signals of divergent selection if at least two sequential windows fell into the 99.9^th^ percentile scores in its heterozygosity’s bin. Preliminary analyses carried out using windows defined based on physical size (1000 Kb, 750 Kb of overlapping) and SNP number (15 and 30) yielded consistent results (Supplementary Fig. S5).

### Genes and QTL identification

Genomic regions identified into the top 99.9 percentile of more than one of the tests (ROH, varLD, and F_ST_) were considered selection signatures, as they are more likely of being true positive signals^16,88^. Known QTL overlapping the selection signatures were assessed in the CattleQTL database - release 40^89^, with QTL positions lifted from the former reference genome UMD3.1.1 to the new reference ARS-UCD 1.2. The Ensembl cow gene - set 99^90^, which is based on the new ARS-UCD1.2 reference, was used for gene prospection.

Two lists of candidate genes were evaluated as over-representation of biological processes and molecular pathways, using the DAVID v.6.7^91^ and PANTHER v.13.1^92^. One list included 26 genes surrounded by ROH islands common for the two subpopulations and a further list included 73 genes of genomic regions divergently selected (ROH islands exclusively detected in one of the two subpopulations, F_ST_ and varLD signals).

## Supplementary information

Supplementary information.

## Data Availability

The datasets used and/or analyzed during the current study are available upon reasonable requests to the corresponding authors.
