# Publisher Correction: Uncovering Sub-Structure and Genomic Profiles in Across-Countries Subpopulations of Angus Cattle

**DOI:** 10.1038/s41598-020-70101-2

**Published:** 2020-10-08

**Authors:** Diercles Francisco Cardoso, Gerardo Alves Fernandes Júnior, Daiane Cristina Becker Scalez, Anderson Antonio Carvalho Alves, Ana Fabrícia Braga Magalhães, Tiago Bresolin, Ricardo Vieira Ventura, Changxi Li, Márcia Cristina de Sena Oliveira, Laercio Ribeiro Porto-Neto, Roberto Carvalheiro, Henrique Nunes de Oliveira, Humberto Tonhati, Lucia Galvão Albuquerque

**Affiliations:** 1 Department of Animal Science, School of Agricultural and Veterinarian Science, São Paulo State University (UNESP), Jaboticabal, SP Brazil; 2 Department of Animal Nutrition and Production, School of Veterinary Medicine and Animal Science (FMVZ), University of Sao Paulo (USP), Pirassununga, SP Brazil; 3 Department of Agricultural, Food and Nutritional Science, Faculty of Agricultural, Life & Environmental Sciences, University of Alberta, Edmonton, AB Canada; 4 Lacombe Research and Development Centre, Agriculture and Agri-Food Canada, Lacombe, AB Canada; 5 Embrapa Pecuária Sudeste, São Carlos, SP Brazil; 6 CSIRO Agriculture & Food, 306 Carmody Rd., St. Lucia, Brisbane, QLD Australia; 7 National Council for Science and Technological Development, Brasília, Distrito Federal Brazil; 8 Present Address: Centre for Genetic Improvement of Livestock, Department of Animal Biosciences, University of Guelph, Guelph, ON Canada

Correction to: *Scientific Reports* 10.1038/s41598-020-65565-1, published online 29 May 2020

In the original version of this Article, Gerardo Alves Fernandes Júnior was incorrectly affiliated with ‘Present address: Centre for Genetic Improvement of Livestock, Department of Animal Biosciences, University of Guelph, Guelph, ON, Canada.’

In addition, an affiliation was omitted for Daiane Cristina Becker Scalez. The correct affiliations are listed below.

Gerardo Alves Fernandes Júnior:

Department of Animal Science, School of Agricultural and Veterinarian Science, São Paulo State University (UNESP), Jaboticabal, SP, Brazil.

Daiane Cristina Becker Scalez:

Department of Animal Science, School of Agricultural and Veterinarian Science, São Paulo State University (UNESP), Jaboticabal, SP, Brazil.

Present address: Centre for Genetic Improvement of Livestock, Department of Animal Biosciences, University of Guelph, Guelph, ON, Canada.

These errors have been corrected in the PDF and HTML versions of the Article.

