# Supplementary material for: Uncovering Sub-Structure and Genomic Profiles in Across-Countries Subpopulations of Angus Cattle
Source: Sci Rep. 2020 May 29;10:8770. doi: 10.1038/s41598-020-65565-1 (PMC7260210; doi:10.1038/s41598-020-65565-1)
Supplement: Supplementary file 1 — Supplementary information. [file 41598_2020_65565_MOESM1_ESM.docx]

**UNCOVERING SUB-STRUCTURE AND GENOMIC PROFILES IN ACROSS-COUNTRIES SUBPOPULATIONS OF ANGUS CATTLE**

Diercles Francisco Cardoso, Gerardo Alves Fernandes Júnior, Daiane Cristina Becker Scalez, Anderson Antonio Carvalho Alves, Ana Fabrícia Braga Magalhães, Tiago Bresolin, Ricardo Vieira Ventura, Changxi Li, Márcia Cristina de Sena Oliveira, Laercio Ribeiro Porto-Neto, Roberto Carvalheiro, Henrique Nunes de Oliveira, Humberto Tonhati and Lucia Galvão Albuquerque


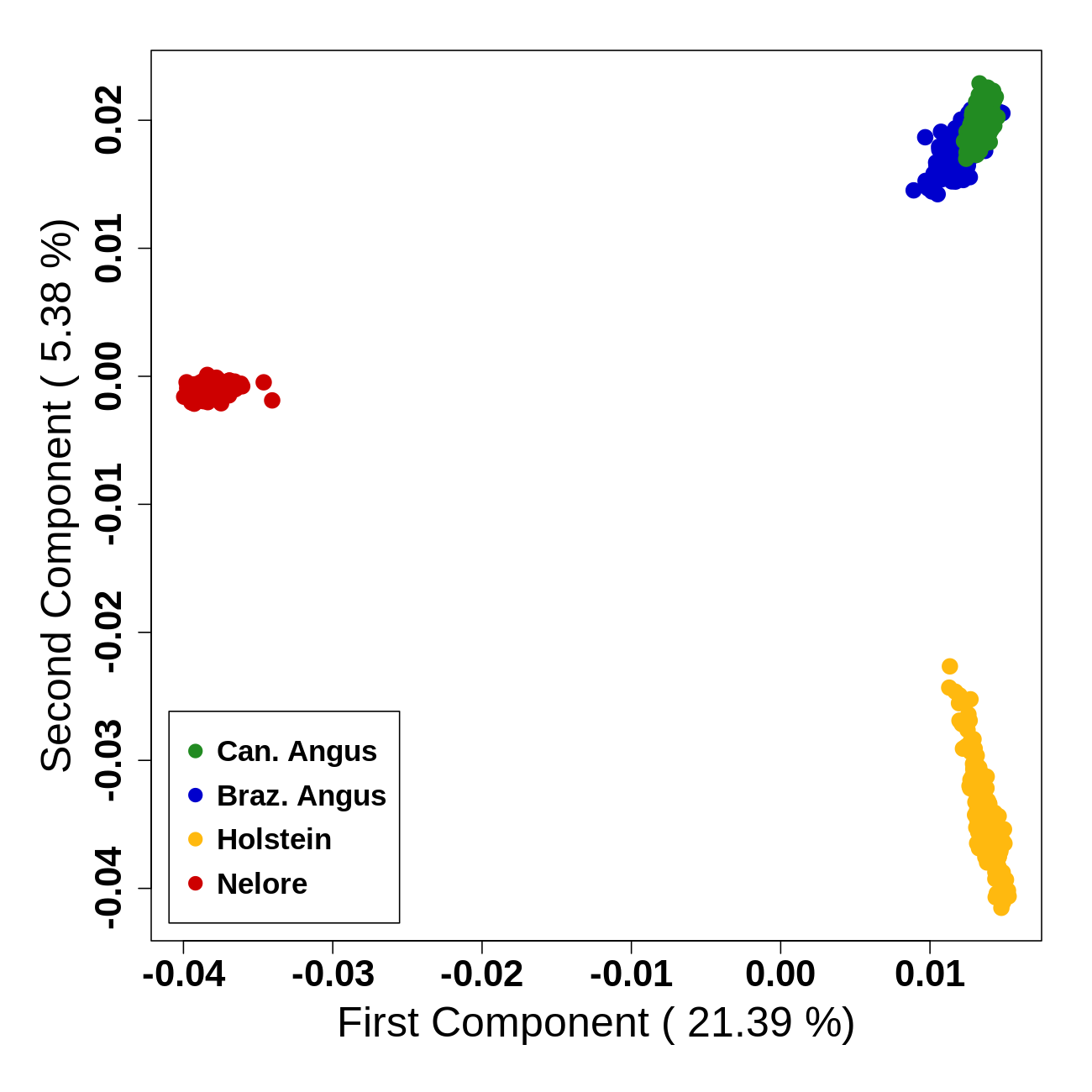


**Supplementary Figure S1. Principal component analysis of genotypes for Brazilian and Canadian subpopulations of Angus and two additional cattle breeds, Holstein and Nelore.**

**Supplementary Table S1.** Distribution of SNPs per chromosome (BTA), number of SNPs (N), mean distance (Mean dist.) and square correlation means (r²) according to different minor allele frequency (MAF) threshold in the Brazilian and Canadian subpopulations.

| BTA | N | Mean dist. (kb) | | | r^2^ | | | | | | |
| --- | --- | --- | --- | --- | --- | --- | --- | --- | --- | --- | --- |
|  | | | | Brazilian Angus^1^ | | | |  | Canadian Angus^1^ | | |
|  |  |  |  | MAF0 | | MAF1 | MAF5 |  | MAF0 | MAF1 | MAF5 |
| 1 | 1,986 | | 79.21 | 0.195 | | 0.199 | 0.215 |  | 0.207 | 0.211 | 0.229 |
| 2 | 1,694 | | 80.21 | 0.195 | | 0.197 | 0.213 |  | 0.205 | 0.206 | 0.222 |
| 3 | 1,525 | | 79.21 | 0.209 | | 0.211 | 0.231 |  | 0.226 | 0.229 | 0.249 |
| 4 | 1,515 | | 78.85 | 0.197 | | 0.198 | 0.216 |  | 0.209 | 0.210 | 0.227 |
| 5 | 1,310 | | 91.60 | 0.193 | | 0.196 | 0.224 |  | 0.209 | 0.212 | 0.240 |
| 6 | 1,521 | | 77.32 | 0.194 | | 0.198 | 0.211 |  | 0.216 | 0.220 | 0.231 |
| 7 | 1,347 | | 81.81 | 0.211 | | 0.212 | 0.228 |  | 0.229 | 0.231 | 0.249 |
| 8 | 1,532 | | 73.45 | 0.199 | | 0.202 | 0.222 |  | 0.209 | 0.213 | 0.232 |
| 9 | 1,266 | | 82.34 | 0.197 | | 0.202 | 0.216 |  | 0.208 | 0.213 | 0.227 |
| 10 | 1,363 | | 74.87 | 0.178 | | 0.181 | 0.191 |  | 0.188 | 0.191 | 0.202 |
| 11 | 1,362 | | 78.42 | 0.197 | | 0.200 | 0.216 |  | 0.214 | 0.217 | 0.234 |
| 12 | 994 | | 87.43 | 0.178 | | 0.182 | 0.191 |  | 0.190 | 0.194 | 0.207 |
| 13 | 1,142 | | 72.69 | 0.214 | | 0.216 | 0.234 |  | 0.233 | 0.235 | 0.254 |
| 14 | 1,130 | | 72.71 | 0.198 | | 0.204 | 0.218 |  | 0.217 | 0.223 | 0.237 |
| 15 | 1,050 | | 80.24 | 0.166 | | 0.169 | 0.185 |  | 0.179 | 0.184 | 0.203 |
| 16 | 1,004 | | 79.67 | 0.198 | | 0.201 | 0.221 |  | 0.210 | 0.212 | 0.232 |
| 17 | 997 | | 73.11 | 0.165 | | 0.167 | 0.176 |  | 0.178 | 0.180 | 0.190 |
| 18 | 816 | | 79.61 | 0.186 | | 0.187 | 0.198 |  | 0.212 | 0.213 | 0.226 |
| 19 | 836 | | 75.39 | 0.173 | | 0.175 | 0.187 |  | 0.191 | 0.193 | 0.205 |
| 20 | 926 | | 76.89 | 0.158 | | 0.160 | 0.172 |  | 0.179 | 0.180 | 0.197 |
| 21 | 846 | | 80.84 | 0.190 | | 0.191 | 0.198 |  | 0.198 | 0.200 | 0.208 |
| 22 | 770 | | 78.69 | 0.170 | | 0.171 | 0.189 |  | 0.186 | 0.187 | 0.207 |
| 23 | 666 | | 78.44 | 0.170 | | 0.173 | 0.178 |  | 0.181 | 0.185 | 0.191 |
| 24 | 764 | | 80.85 | 0.190 | | 0.196 | 0.211 |  | 0.209 | 0.214 | 0.228 |
| 25 | 622 | | 67.89 | 0.176 | | 0.178 | 0.189 |  | 0.190 | 0.191 | 0.204 |
| 26 | 689 | | 74.46 | 0.173 | | 0.174 | 0.195 |  | 0.184 | 0.186 | 0.206 |
| 27 | 590 | | 76.93 | 0.175 | | 0.181 | 0.195 |  | 0.185 | 0.191 | 0.208 |
| 28 | 583 | | 77.64 | 0.158 | | 0.159 | 0.165 |  | 0.180 | 0.182 | 0.188 |
| 29 | 637 | | 79.20 | 0.153 | | 0.156 | 0.163 |  | 0.163 | 0.166 | 0.173 |

^1^Minor allele frequency (MAF) criteria: MAF0 = (MAF > 0%) ; MAF1: (MAF > 1%); MAF5: (MAF > 1%)

**Supplementary Table S2.** **ROH islands identified in Brazilian and Canadian Subpopulation of Angus and candidate genes.**

| **Genomic Regions^1^** | **Subpopulations** | **Genes** |
| --- | --- | --- |
| BTA7:37.68-38.64 | Canadian | *COMMD10, ARL10, NOP16, HIGD2A, CLTB, FAF2, RNF44, CDHR2, GPRIN1, SNCB, EIF4E1B, TSPAN17, UNC5A, HK3, UIMC1, ZNF346, FGFR4, NSD1* |
| BTA13:63.77-65.25 | Brazilian | *ITCH, DYNLRB1, MAP1LC3A, PIGU, TP53INP2, NCOA6, GGT7, ACSS2, GSS, MYH7B, TRPC4AP, EDEM2, PROCR, MMP24, EIF6, FAM83C, UQCC1, GDF5, GDF5OS, CEP250, ERGIC3, SPAG4, CPNE1, RBM12, NFS1, ROMO1, RBM39, PHF20, SCAND1, CNBD2, EPB41L1* |
| BTA13:63.77-65.25 | Canadian | *ITCH, DYNLRB1, MAP1LC3A, PIGU, TP53INP2, NCOA6, GGT7, ACSS2, GSS, MYH7B, TRPC4AP, EDEM2, PROCR, MMP24, EIF6, FAM83C, UQCC1, GDF5, GDF5OS, CEP250, ERGIC3, SPAG4, CPNE1, RBM12, NFS1, ROMO1, RBM39, PHF20, SCAND1, CNBD2, EPB41L1* |
| BTA14:22.81-24.72 | Brazilian | *XKR4, TMEM68, TGS1, LYN, RPS20, MOS, PLAG1, CHCHD7, SDR16C5, SDR16C6, PENK, IMPAD1, FAM110B, UBXN2B, CYP7A1* |
| BTA14:22.81-23.57 | Canadian | *XKR4, TMEM68, TGS1, LYN, RPS20, MOS, PLAG1, CHCHD7, SDR16C5, SDR16C6, PENK* |

^1^Chromosome:StartPosition(Mb):EndPosition(Mb) (based on assembly ARS-UCD1.2)

**Supplementary Table S3.** **Genomic regions spanning the top 99.9 percentile of FST and varLD window scores in the comparisons between Canadian and Brazilian subpopulations of Angus.**

| **Genomic Regions^1^** | **Method** | **Genes** |
| --- | --- | --- |
| BTA7:21.30-21.89 | varLD | *DOT1L, PLEKHJ1, SF3A2, AMH, JSRP1, AP3D1, IZUMO4, MOB3A, MKNK2, SEPTIN8, CCNI2, KIF3A, IL4, IL13, RAD50, IL5* |
| BTA7:21.31-22.11 | F_ST_ | *DOT1L, PLEKHJ1, SF3A2, AMH, JSRP1, AP3D1, IZUMO4, MOB3A, MKNK2, SEPTIN8, CCNI2, KIF3A, IL4, IL13, RAD50, IL5, IRF1, SLC22A5, SLC22A4* |
| BTA7:37.84-38.36 | F_ST_ | *ARL10, NOP16, HIGD2A, CLTB, FAF2, RNF44, CDHR2, GPRIN1, SNCB, EIF4E1B, TSPAN17, UNC5A, HK3, UIMC1* |
| BTA11:22.38-22.54 | F_ST_ | *SLC8A1* |
| BTA11:70.69-70.91 | varLD | *ALK* |
| BTA18:11.48-11.94 | varLD | *GSE1, GINS2, EMC8, COX4I1, IRF8* |
| BTA18:11.75-12.09 | F_ST_ | *EMC8, COX4I1, IRF8* |
| BTA18:13.65-15.46 | F_ST_ | *ZFPM1, qZC3H18, CYBA, MVD, SNAI3, CTU2, RNF166, PIEZO1, CDT1, APRT, GALNS, TRAPPC2L, CBFA2T3, ACSF3, CDH15, SLC22A31, ANKRD11, SPG7, RPL13, CPNE7, DPEP1, CHMP1A, CDK10, SPATA2L, VPS9D1, ZNF276, FANCA, SPIRE2, TCF25, MC1R, TUBB3, DEF8, DBNDD1, GAS8, SHCBP1, VPS35, ORC6, MYLK3, C18H16orf87, GPT2, DNAJA2* |
| BTA18:13.76-15.40 | varLD | *ZFPM1, qZC3H18, CYBA, MVD, SNAI3, CTU2, RNF166, PIEZO1, CDT1, APRT, GALNS, TRAPPC2L, CBFA2T3, ACSF3, CDH15, SLC22A31, ANKRD11, SPG7, RPL13, CPNE7, DPEP1, CHMP1A, CDK10, SPATA2L, VPS9D1, ZNF276, FANCA, SPIRE2, TCF25, MC1R, TUBB3, DEF8, DBNDD1, GAS8, SHCBP1, VPS35, ORC6, MYLK3, C18H16orf87, GPT2, DNAJA2* |
| BTA27:16.19-16.59 | varLD | *FAM149A, CYP4V2, KLKB1, F11, MTNR1A, FAT1* |

^1^Chromosome:StartPosition(Mb):EndPosition(Mb)


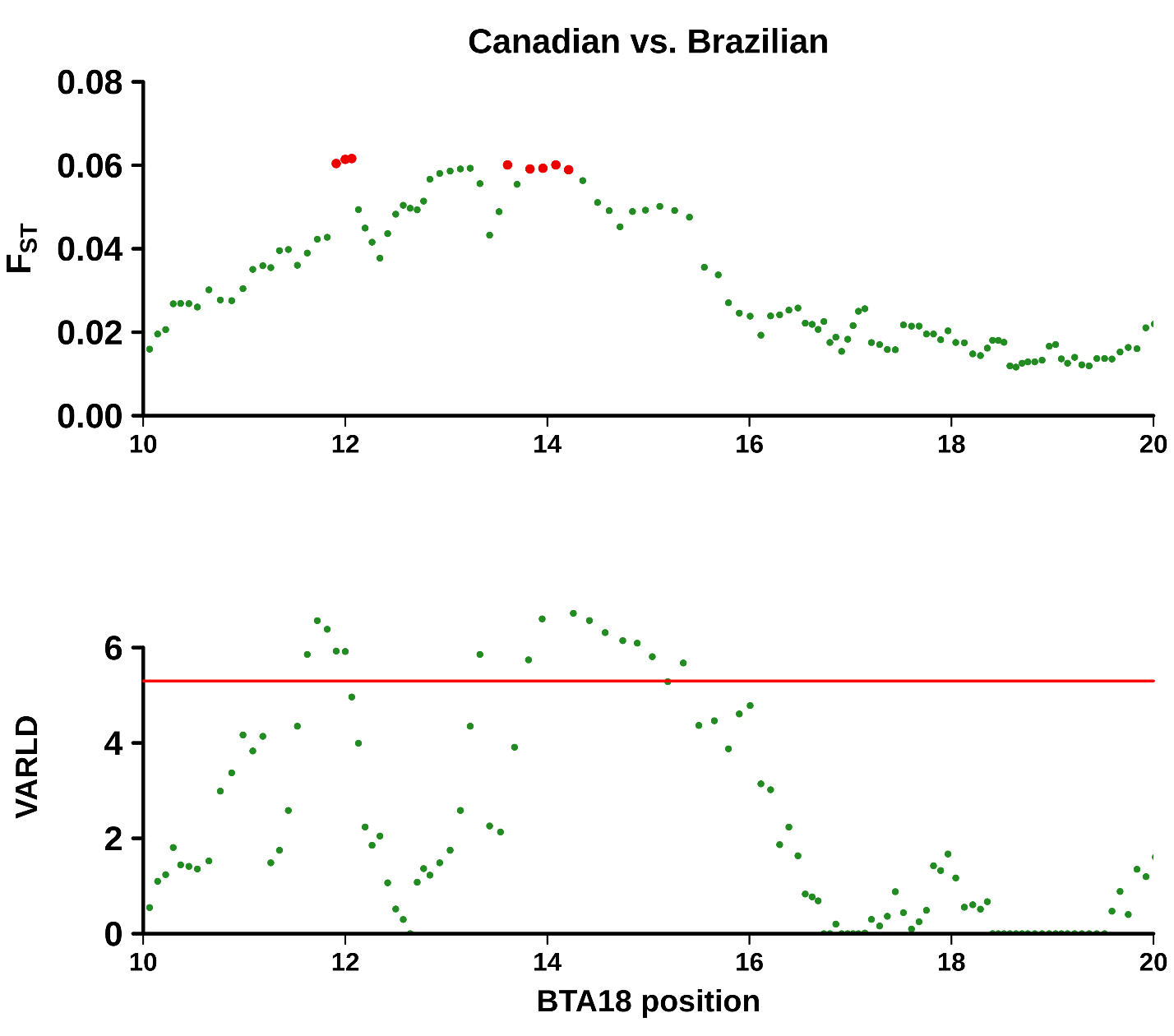


**Supplementary Figure S2. F_ST_ (upper) and varLD (lower) scores across 10 to 20 Mb of the BTA18, with 382 and 360 black Angus from Canada and Brazil, respectively.** Red dots represent F_ST_ windows in 99.9 percentile of their heterozygosity bins. Red lines indicate the cutoff of 99.9 percentile to varLD scores.


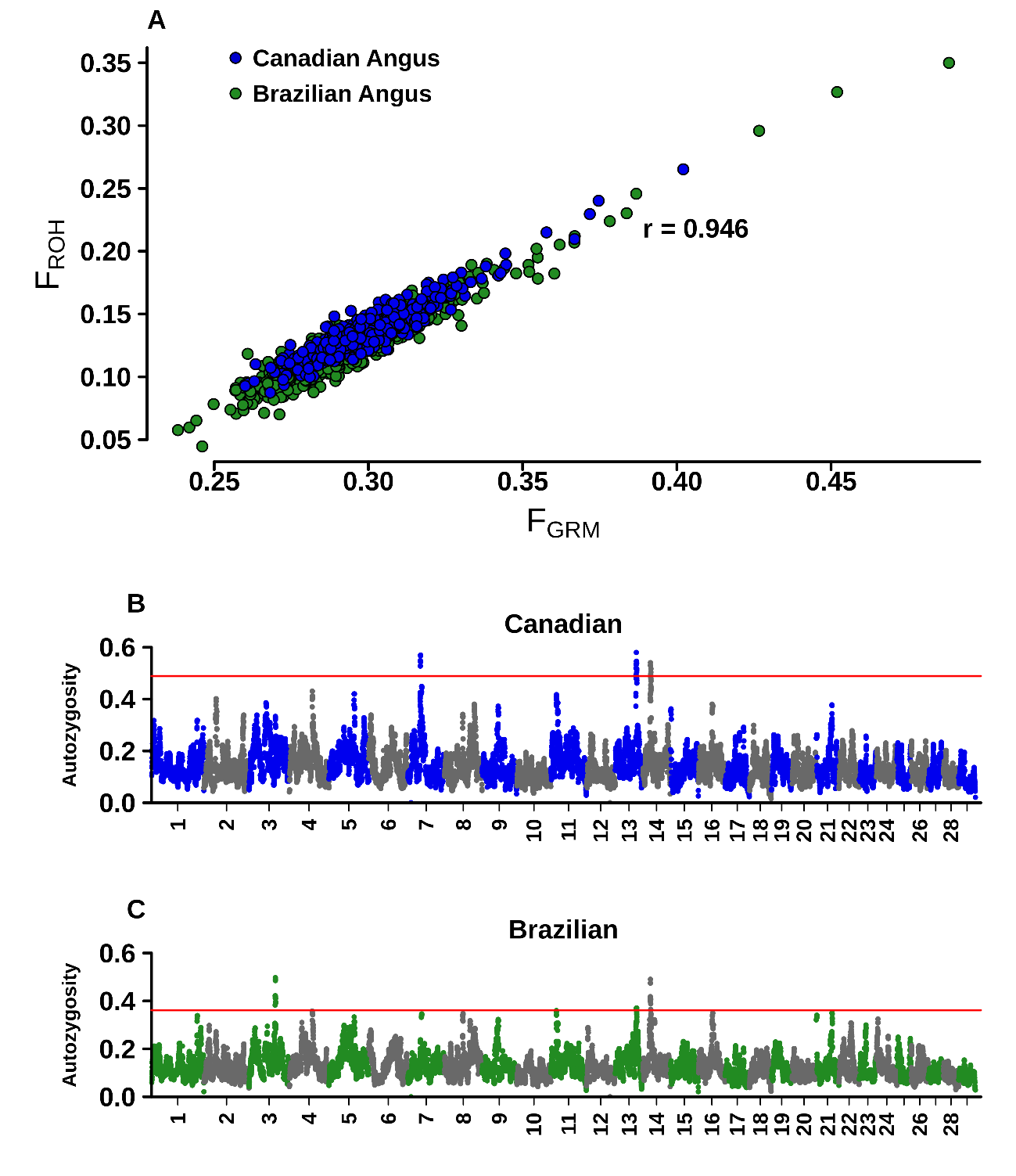
**Supplementary Figure S3. Runs of homozygosity based on consecutive SNPs approach of the tool detectRUNS.** (A) Correlation of genomic inbreeding estimated through runs of homozygosity (F_ROH_) and genomic relationship matrix (F_GRM_). (B and C) Autozygosity scores per SNP within Canadian and Brazilian subpopulations.

**Supplementary Table S4. Autozygosity islands detected with the consecutive SNP-based run detection in the package detectRUNS.**

| **Subpopulation** | **Genomic Regions** |
| --- | --- |
| Brazilian | BTA3:78.53.-80.23 |
| Brazilian | BTA13:63.77-65.09 |
| Brazilian | BTA14:22.35-23.98 |
| Canadian | BTA7:37.68-38.75 |
| Canadian | BTA13:63.36-64.62 |
| Canadian | BTA14:22.81-24.53 |

^1^Chromosome:StartPosition(Mb):EndPosition(Mb)

**Supplementary Figure S4. Comparison of varLD results across**
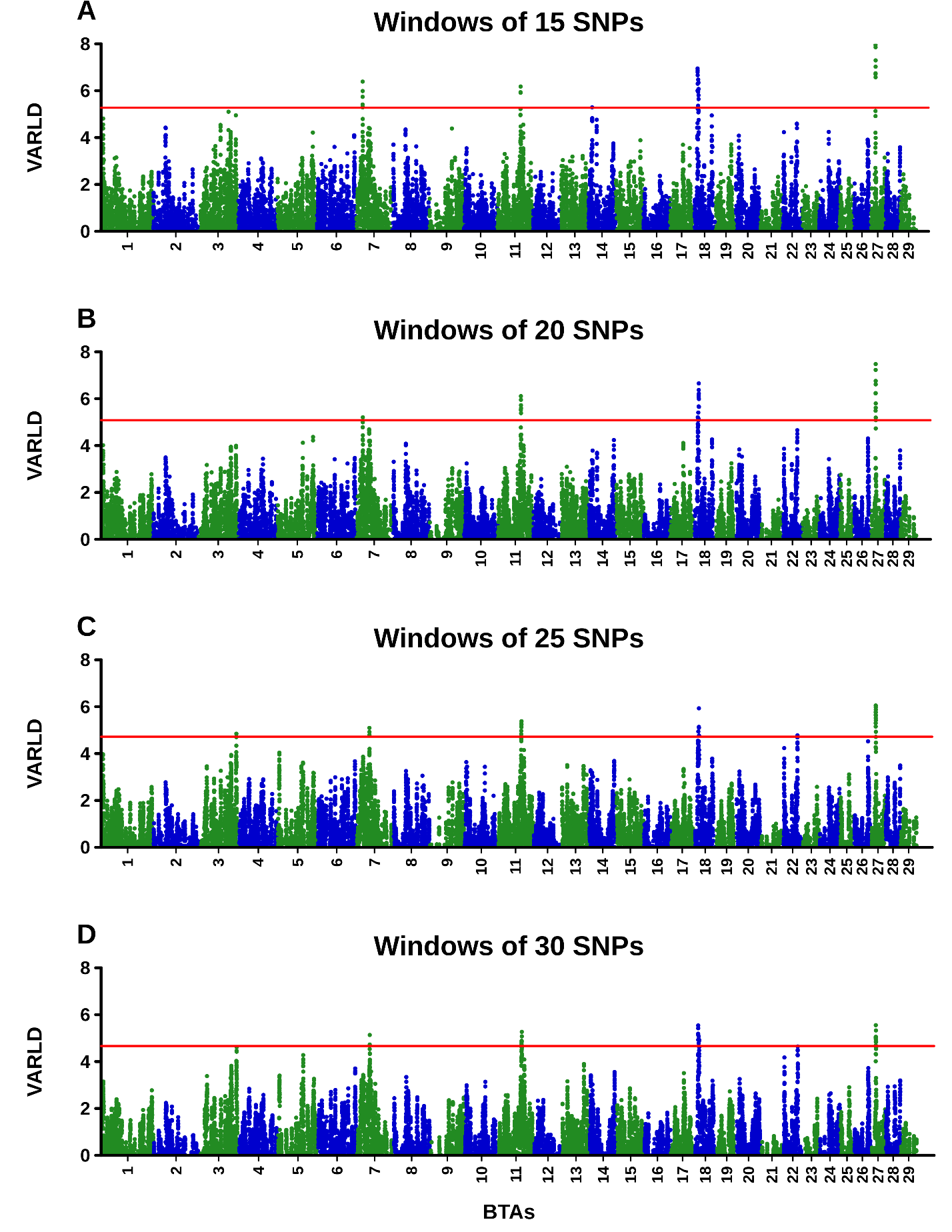
**windows with different number of SNPs.**

**
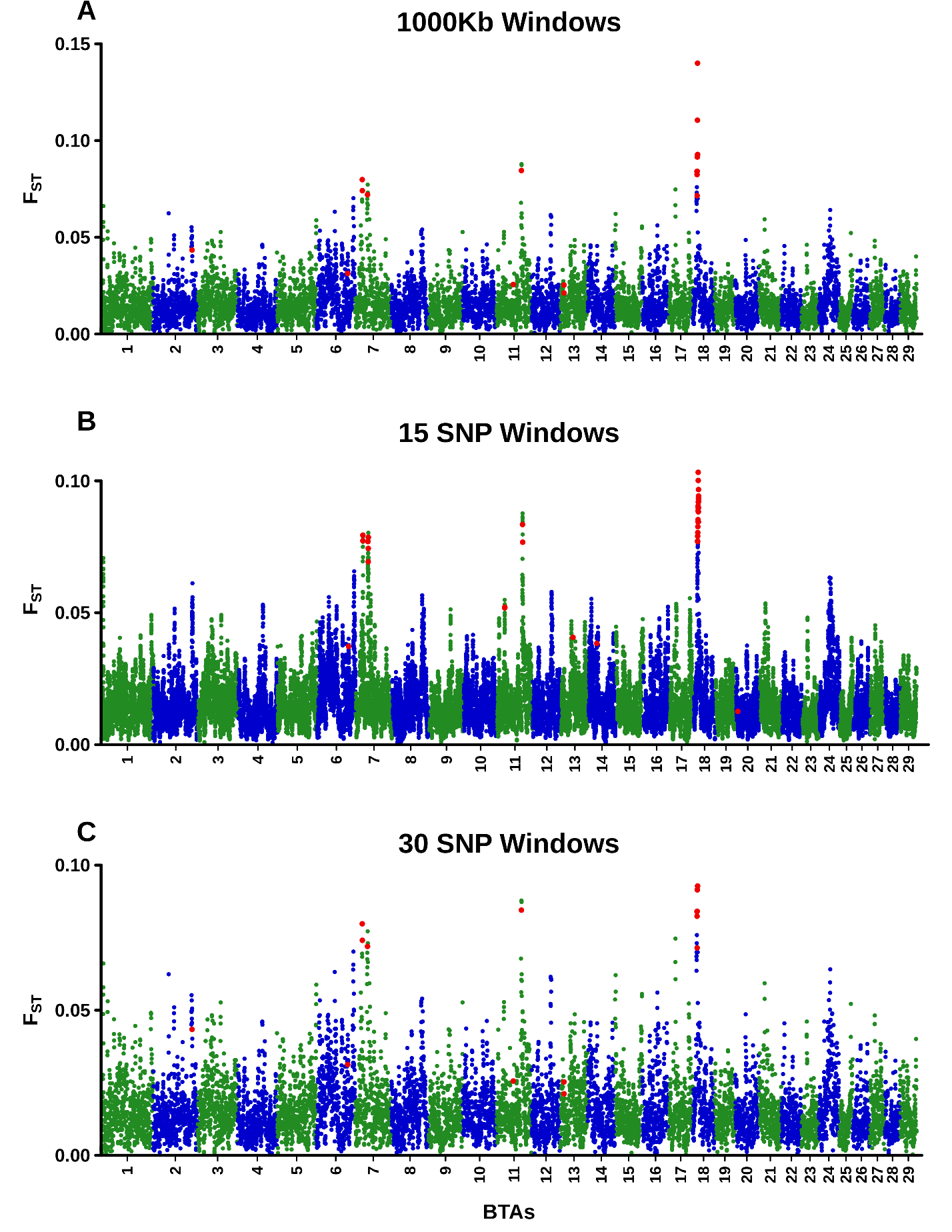
**

**Supplementary Figure S5. Comparison of F_ST_ results across different windows.** Red dots represent F_ST_ windows in 99.9 percentile of their heterozygosity bins.
